# Enhancing breakpoint resolution with deep segmentation model: A general refinement method for read-depth based structural variant callers

**DOI:** 10.1371/journal.pcbi.1009186

**Published:** 2021-10-11

**Authors:** Yao-zhong Zhang, Seiya Imoto, Satoru Miyano, Rui Yamaguchi

**Affiliations:** 1 Division of Health Medical Intelligence, Institute of Medical Science, the University of Tokyo, Tokyo, Japan; 2 M&D Data Science Center, Tokyo Medical and Dental University, Tokyo, Japan; 3 Division of Cancer Systems Biology, Aichi Cancer Center Research Institute, Nagoya, Japan; 4 Division of Cancer Informatics, Nagoya University Graduate School of Medicine, Nagoya, Japan; Princeton University, UNITED STATES

## Abstract

Read-depths (RDs) are frequently used in identifying structural variants (SVs) from sequencing data. For existing RD-based SV callers, it is difficult for them to determine breakpoints in single-nucleotide resolution due to the noisiness of RD data and the bin-based calculation. In this paper, we propose to use the deep segmentation model UNet to learn base-wise RD patterns surrounding breakpoints of known SVs. We integrate model predictions with an RD-based SV caller to enhance breakpoints in single-nucleotide resolution. We show that UNet can be trained with a small amount of data and can be applied both in-sample and cross-sample. An enhancement pipeline named RDBKE significantly increases the number of SVs with more precise breakpoints on simulated and real data. The source code of RDBKE is freely available at https://github.com/yaozhong/deepIntraSV.

## Author summary

In this paper, we used the deep segmentation model UNet to alleviate the bin size limitation of RD-based SV callers. UNet was initially proposed for image data. Here, we demonstrated that the UNet model could also be applied for one-dimensional genomic data. We formalized the breakpoint prediction as a segmentation task and inferred breakpoints in single-nucleotide resolution from predicted label marks. Through a set of experiments on both simulated and real WGS data, we demonstrated that the UNet model could be trained with a small amount of data, and an enhancement pipeline called RDBKE significantly increased the number of SVs with more precise breakpoints.

This is a *PLOS Computational Biology* Methods paper.

## Introduction

Structural variants (SVs) are genomic alterations with lengths greater than 50 base pairs (bp). Compared with small-size mutations, such as single nucleotide polymorphisms (SNPs) and InDels, the larger size of SVs makes them more likely to alter genome structures and have functional consequences. In many diseases, such as Alzheimer’s disease and cancer, SVs have been found to play important roles [1–4]. With advances in sequencing technologies, a more accurate and comprehensive profiling of SVs on a whole-genome scale becomes available.

For detecting SVs from whole-genome sequencing (WGS) data, various algorithms have been developed. (Here, copy number variations (CNVs) are treated as a subtype of SVs). In general, those methods can be classified into four major categories [5]: read-depth (RD) based [6], paired-end (PE) mapping based [7], split-read (SR) based [8] and de novo assembly (AS) based [9]. RD-based methods first divide a reference genome into non-overlapping bins and calculate an RD for each bin. Duplications (DUPs) and deletions (DELs) are detected based on abnormal RD changes of adjacent bins. PE-based methods use span and orientation of paired-end reads, detecting more SV types, such as inversions (INVs). For SR-based methods, if an adjoining read is separately aligned to different coordinates of a reference genome, the alignment can be used to determine breakpoints of an SV in single-nucleotide resolution. AS-based methods assemble reads into larger contigs and use the assembly contigs to detect SVs. However, the assembly itself has a high computational cost for short-read sequencing data. Besides the above four categories, several methods integrate more than one method. For example, DELLY [7] integrated both PE and SR for SV detection. Recent benchmark studies [10, 11] show that no single SV caller can accurately and sensitively detect all types and all sizes of SVs.

In this paper, we focus on RD-based methods. The bin size is an important parameter that affects the performance of RD-based SV callers. A large bin size is reliable to capture large SVs, such as CNVs. However, the large bin size brings about a sensitivity loss for detecting small-length SVs. On the other hand, calculating RD using a small bin size is very noisy and sparse, which results in more false positives. Here, we use the deep segmentation model UNet to enhance the breakpoint resolution of SVs. More specifically, we train the UNet model to learn base-wise RD patterns surrounding breakpoints of known SVs and apply the model to segment RD vectors including breakpoints of candidate SVs. We infer new breakpoints in single-nucleotide resolution from the segmentation results. Compared with the recent work on applying deep learning methods for genotype profiling, our method has two novel points. First, the breakpoint detection is formalized as a segmentation task. Usually, deep learning models are used as classifiers. For instance, DeepVariant [12] uses a well-calibrated convolutional neural network to call SNPs and InDels based on the image pileups of putative sites. Second, our proposed method leverages the power of traditional approaches and deep learning methods. We first use an RD-based SV caller to predict initial breakpoints in bin resolution. Then we apply the segmentation model to screen base-wise RDs surrounding candidate breakpoints for further refinement. We conduct a series of experiments on both simulated and real WGS data. For the real data, we perform systematic experiments both in-sample and cross-sample. The experiment results show that the UNet model can be trained with a small amount of data and can be applied both in-sample and cross-sample. The proposed pipeline using UNet significantly increases the number of SVs with more precise breakpoints.

## Methods and materials

### Overall pipeline of breakpoint enhancement

**Fig 1:**
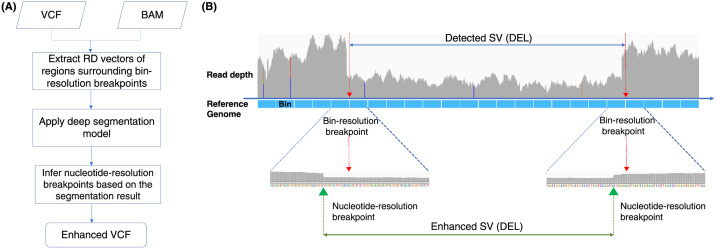
(A) Overall pipeline of RDBKE for enhancing an RD-based SV caller with a deep segmentation model. The initial SV predictions in bin resolution are provided as a VCF file. (B) Example illustrating the enhancement of bin-resolution breakpoints using RDs surrounding breakpoint candidates.

Fig 1A introduces the overall pipeline of RDBKE for enhancing breakpoints of an RD-based SV caller to single-nucleotide resolution. Before enhancement, an RD-based SV caller predicts initial SVs with breakpoints in bin resolution. For a typical RD-based SV caller (e.g., CNVnator [6]), reads are first aligned to a reference genome. The reference genome is split into non-overlapping bin regions with a fixed length. The number of mapped reads covered by a bin is counted as a smoothed RD of the bin. The bin size of an RD-based SV caller affects the sensitivity of the SV caller and determines the resolution of SV’s breakpoints. In the enhancement stage, the genomic regions surrounding candidate breakpoints are further analyzed by a deep segmentation model, which identifies SV overlapping regions inside a screening window. The new breakpoints are then inferred based on SV overlapping marks and updated accordingly, as shown in Fig 1B.

We apply the enhancement pipeline in the following two scenarios. One is the in-sample application. For a sample, we assume a small amount of SVs is validated. These known SVs are used to train or tune the UNet model. Then, the trained UNet model is applied to refine the rest of the non-validated SVs in the sample. The other is the cross-sample application. For those samples sequenced on the same platform, we train the UNet model on the comprehensively investigated sample (e.g., NA12878) and enhance the others.

### UNet segmentation model

In general, regular base-wise RDs are treated as too noisy to be directly used in determining SVs’ breakpoints. In fact, with a proper design of the model structure, a deep learning model can be used to process base-wise RD data directly and learn to recognize RD patterns surrounding candidate breakpoints. Here, we use a deep segmentation model to label SV overlapping coordinates and infer breakpoint positions from the segmentation results.

**Fig 2:**
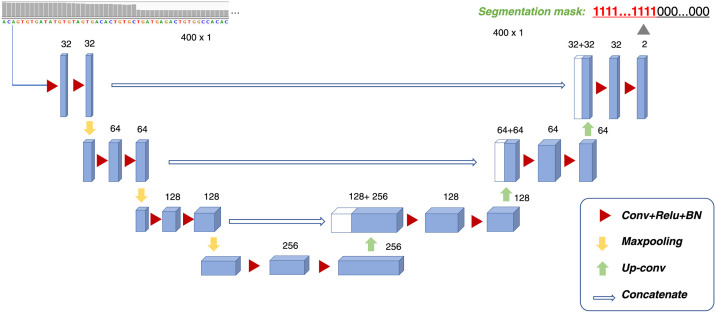
Model structure of the deep segmentation model UNet used for labeling SV overlapping coordinates.

Formally, given the base-wise RD vector *X* = {*d*_1_, *d*_2_, …, *d*_*l*_} of a screening window of *l* bp, we aim to find the segmentation *Y* = {*m*_1_, *m*_2_, …, *m*_*l*_}, in which *m*_*i*_ ∈ {0, 1} indicates whether the coordinate *i* overlaps with an SV. We use UNet [13] to learn the mapping from *X* to *Y*. UNet is a deep neural network featured with its U-shaped architecture. It combines the advantages of the convolutional neural network (CNN) and the auto-encoder (AE). In image segmentation tasks, especially in medical image segmentation tasks, it achieves the state-of-the-art performance [14]. The structure of UNet is described in Fig 2. It is an encode-and-decode architecture consisting of the left encoding module and the right decoding module. The left encoding module denoises base-wise RD signals and extracts RD features by repeatedly applying a convolutional layer followed by a rectified linear unit (ReLU) and a batch normalization (BN). A max-pooling operation is applied in every two module blocks. The right-U expands the down-sampled feature map by up-convolution and concatenates the feature map of the corresponding layer in the left-U module. The skip-connection between down-sampled and up-sampled tensors is designed to avoid gradient vanishes and maintain positional information. Two additional convolution layers follow each concatenated feature map with ReLU and BN. In the output layer, 1x1 convolution is applied with the *Sigmoid* active function to predict label marks. Compared with a typical CNN model, there is no fully connected layer in UNet.

For further inferring breakpoint positions from segmentation results, we let the output of UNet has the same length as the input. We use padding and select proper max-pooling size to avoid non-divisible numbers of hidden units during the down-sampling and up-sampling process. The label of each position is predicted based on a criterion of whether the prediction score is greater than 0.5. If it is greater than 0.5, label “1” is assigned to indicate the position overlaps with an SV. Or else, label “0” is predicted.

### Training the UNet model

To train the UNet model, we utilize validated SVs and generate base-wise RD vectors surrounding known breakpoints as positive samples. All-zero RD vectors are filtered out. Note that RD-based SV callers can only predict DELs and DUPs. As a breakpoint may not locate in the center position of a screening window, we add a random shift to the start position of the screening window. The distance between the start position and the breakpoint is randomly sampled in the range of [10, *l*-10]. A region with too small SV overlapping (the length of nucleotides overlap with an SV is less than 10 bp) is treated as too difficult to make proper predictions only with the RD information. These samples are excluded from the training set. We generate the same amount of randomly selected RD vectors from the rest genomic regions without any reported SV as the negative samples.

We use Dice similarity coefficient (DSC) [15] as the loss function, and use Adam [16] as the optimizer to train the UNet model.

$$\begin{array}{c} DSC\left(Y_{pred},Y_{gold}\right)=\frac{2\sum_{i=1}^{l}m_{gold}^{i}*m_{pred}^{i}+\epsilon }{\sum_{i=1}^{l}m_{gold}^{i}+\sum_{i=1}^{l}m_{pred}^{i}+\epsilon }, \end{array}$$

where *m*_*gold*_ and *m*_*pred*_ are the gold-standard (GS) and predicted label marks, and *ϵ* (e.g., 1e-7) is a numeric tolerance to avoid division by zero.

### Using segmentation results for refining breakpoints of SVs

We use Algorithm 1 to infer breakpoint positions from segmentation results. As the enhancement is independently performed for each breakpoint of an SV, we take an extra check on the enhanced SVs, whether their SV sizes are less than 50 bp. If the enhancement makes an SV shorter than 50 bp, we retain the original SV boundary.

### Evaluation data

We used the simulated data provided by Kosugi et al. [10], which was designed for systematic benchmarking of different SV callers. They predefined SVs with precise breakpoints based on the Database of Genomic Variants (DGV) and validated SVs from the benchmark sample NA12878. The predefined SV set consists of 3530 DELs, 1656 DUPs, 2819 INSs, and 309 INVs. We evaluated DELs and DUPs in autosomes. Paired-end reads (125 bp read length and 500 bp insert length, averaging 30x coverage) were generated based on the simulated GRCh37 human diploid genome with these predefined SVs, using ART simulator [17].

**Algorithm 1: Breakpoint enhancement for SVs**

**1 Input**: SVs predicted by an RD-based SV caller.

**2 Output**: SVs with enhanced breakpoints.

**3 foreach** *SV* ∈ *SVs* **do**

**4**  Extracting read-depth (RD) vectors surrounding the left and right boundary of the SV;

**5**  **foreach** *RD vector* **do**

**6**   Predict SV label marks using a deep segmentation model;

**7**   Generate breakpoint candidates based on the label marks;

**8**   **switch** *number of breakpoint candidates n* **do**

**9**    **case** *n*=*1* **do**

**10**     update breakpoint with the candidate;

**11**    **end**

**12**    **case** *n* > *1* **do**

**13**     use the one closest to the original breakpoint as the new breakpoint;

**14**    **end**

**15**    **otherwise do**

**16**     retain the original breakpoint;

**17**    **end**

**18**   **end**

**19**  **end**

**20 end**

To evaluate the proposed method on real data, we used WGS data from the 1000 Genomes Project (1kGP) and the Genome in a Bottle Consortium (GiaB). Samples with high-quality SV callsets, such as NA12878 and HG002, were included. Besides, NA19238 and NA19239 from 1kGP were used for the cross-sample evaluation. The gold-standard SVs of NA12878, NA19238, NA19239 were generated based on the VCF file (v8) of merged SVs from 1kGP. The gold-standard SVs of HG002 are from the high-confidence Tier1 callset and were filtered with “HG002_count ≥ 8”. More detailed information of VCF files and alignment BAM files are listed in S1 Table.

We normalized RD vectors using the mean and standard derivation of base-wise RDs from randomly sampled bins in the “background” regions (known-SV-overlapping regions and low-mappability regions were excluded) for each sample.

### Evaluation metrics

We evaluated the proposed method in two aspects: model level and application level. At the model level, we evaluated different models by the segmentation and classification tasks. We compared UNet with two commonly used models: CNN and support vector machine (SVM). CNN was used to compare different deep learning models for segmentation, while SVM was applied for the simplified task of classifying whether a screening window contains SV-overlapping regions. To evaluate UNet and CNN in the classification task, we first segmented the screening window’s RD vectors and made classification predictions based on the segmentation results. If there is any SV overlapping mark inside the screening window, it is classified as positive and vice versa. We measured classification performance using metrics of F1 score, FDR, precision, and recall. We used DSC for measuring segmentation performance.

At the application level, we applied breakpoint enhancement using different segmentation models for an RD-based SV caller. We evaluated SV and breakpoint changes before and after the enhancement. Without loss of generality, we used CNVnator as an instance. Other RD-based SV callers can also be used. To evaluate the accuracy of SVs, we used Jaccard similarity (JS) between a predicted SV and a gold-standard SV, which is calculated as follows:

$$\begin{array}{c} J\left(SV_{p},SV_{g}\right)=\frac{|SV_{p}\cap SV_{g}|}{|SV_{p}\cup SV_{g}|}=\frac{l_{overlap}}{l_{gold}+l_{predict}-l_{overlap}}, \end{array}$$

where *l*_*overlap*_ is the length of the overlapping region of the predicted SV and the gold-standard SV. We defined the gold-standard overlapping SV as the one with the JS score greater than 0.5. Compared with reciprocal overlap (RO) used in other related works, the value of JS is less than or equal to that of the RO for the same pair (the denominator of JS is equal to or larger). In other words, JS greater than 0.5 is a relatively stricter metric than the RO greater than 0.5. We calculated the number of gold-standard-overlapping SVs and SVs with precise boundaries (both-boundary-match and partial-boundary-match (left or right)). Here, a “precise” boundary is defined as within 1 bp distance to the gold standard. Besides the evaluation of SVs, we also investigated the number of breakpoints of different precision. We compared the numbers of breakpoints in different distance ranges before and after the enhancement.

### SV callers and model settings

We utilized three commonly used SV callers, CNVnator (v 0.4), Delly (v 0.8.1) and Lumpy (v 0.2.13). CNVnator is an RD-based SV caller, while Delly and Lumpy were used as SV callers of other types for comparison. CNVnator and Delly were run in their default settings. For CNVnator, different bin sizes range from 50 bp to 1000 bp were evaluated on the simulated data. Lumpy was used with the “express” mode for analysis.

For the enhancement models, the default length of the screening window was 400 bp. The model structure of UNet is described in Fig 2. CNN used the typical LeNet structure [18]. More detailed information on network structures can be found in S2 Table. We trained deep learning models using one Nvidia V100 GPU card. Hyper-parameters were determined based on a random training split of the simulated data using Hyperopt (v 0.2). The learning rate and batch size were 0.001 and 64, respectively. We applied early stopping with a minimum of 10 epochs and a maximum of 100 epochs for the training process. SVM used the default setting of the Scikit-learn (v 0.22.2) package.

## Results

### Evaluation on simulated data

We first evaluated the proposed method on the simulated data. Typically, n-fold cross-validation uses *n* − 1 folds for training, while the rest one fold is used for testing. Besides the standard-setting, we also used one fold for training and the other *n* − 1 folds for testing to evaluate models using a small amount of training data.

#### Model level performance

The typical 5-fold cross-validation setting (Train-data-proportion is 80% of total SVs) was first investigated. As shown in Table 1, for the segmentation task, UNet achieves a better segmentation performance with higher DSC scores. More specifically, for the test set containing background samples, DSC-ALL of UNet is 2.61% absolute higher than that of CNN. For the test set only containing SV samples, DSC-BK of UNet is around 2.03% higher than that of CNN. In the binary classification task, UNet achieves the best performance among the three models. UNet has higher scores of F1, precision, and recall while retaining a lower FDR value. For deep segmentation models, UNet and CNN achieve recalls over 85%, which are higher than the recall of SVM, around 81.3%. Compared with CNN, SVM also has a lower FDR value.

**Table 1:** 5-fold cross-validation on the simulated data. The average result of 5-repeat runs is reported to reduce the effect of the randomness of GPU training. The results of repeat runs are shown in S3 Table.

Model	Train-data proportion	Segmentation		Classification			
		DSC-ALL	DSC-BK	F1 score	Precision	Recall	FDR
SVM	80%	/		0.8590	0.9102	0.8133	0.0898
	20%	/		0.8502	0.9002	0.8057	0.0998
CNN	80%	0.7979	0.827	0.8733	0.8962	0.8518	0.1038
	20%	0.7478	0.7882	0.8407	0.8562	0.8266	0.1438
UNet	80%	**0.8240**	**0.8473**	**0.8900**	**0.9288**	**0.8546**	**0.0712**
	20%	0.8030	0.8311	0.8787	0.9121	0.8486	0.0879

#### UNet can be trained with a small amount of data

In real applications, known validated SVs are relatively few. It is worthwhile investigating training deep segmentation models with only a small amount of data. Therefore, we adjusted the data-split setting and used only one fold data for training (Train-data-proportion:20%). For the simulated data of a total of 19974 RD vectors, we trained models on 3989 RD vectors and tested on the rest.

Compared with the model performance in the typical data-split setting (80% Training, 20% Testing), the performance of all the three models decreased when using a smaller amount of training data. As shown in Table 1, in the segmentation task, DSC-BK of UNet has a relative decrease of 1.9%, while DSC-BK of CNN has a relative decrease of 4.7%. In the classification task, the relative decreases of the F1 score for UNet, CNN, and SVM are around 1%, 3.7%, and 1.2%, respectively. CNN is more sensitive to the data amount than the other two models on the data. Nevertheless, the decreases are not as significant as the reduction of training data for nearly three quarters. This result indicates that it is feasible to train the UNet model with a small amount of data.

#### Enhancement of breakpoint resolution

At the application level, we performed enhancement in the following two scenarios. One is the in-sample enhancement that trains a segmentation model with a small amount of validated SVs and does the breakpoint enhancement for the rest of the candidate SVs in the same sample. The other is the cross-sample enhancement, which trains a segmentation model with validated SVs from one sample and enhances SVs of other samples. For the cross-sample case, we assumed both samples are sequenced on the same platform, which is required to maintain models’ generalization ability.

We performed in-sample enhancement on the simulated data. For each sample, we randomly sampled 20% of SVs and trained models. These 20% SVs were excluded from the evaluation. CNVnator was applied to generate initial breakpoints of SVs in bin resolution. The deep segmentation models analyzed the RD of each coordinate in the screening window surrounding these breakpoints. Based on the segmentation results, enhancement breakpoints were generated using Algorithm 1. Table 2 shows the number of specific SVs before and after enhancement. We applied CNVnator using five different bin sizes ranging from 50 bp to 1000 bp. CNVnator predicted more SVs when using a smaller bin size, which is shown in the “predicted SVs” column of Table 2. As the read-coverage of WGS data is fixed, the smaller the bin size is, the fewer reads are counted in a bin. Therefore, bin-based RDs become sparse and noisy as the bin size decreases, especially when the bin size is less than 100 bp. For CNVnator, the number of predicted SVs has increased 21.3% to 4766 as the bin size reduces from 100 bp to 50 bp. However, the number of gold-standard overlapping SVs does not increase in proportion to the increased predictions, which indicates the CNVnator using a smaller bin size tends to have more false-positive predictions.

**Table 2:** Enhancement of the predicted SVs by CNVnator using different bin sizes. The length of the screening window surrounding predicted breakpoints is 400 bp. “GS-ov” SVs are referred to as gold-standard overlapping SVs. “l/r match” represents SVs with partial-boundary-match (left or right), and “l&r match” denotes SVs with both-boundary-match (left and right).

CNVnator			Specific SVs w/wo Enhancement			
Bin size	predicted SVs	enhancement	GS-ov SVs	(1) l/r match	(2) l&r match	$\frac{\left(1)+(2\right)}{\text{GS-ov}\;\text{SVs}}$
50	4766	/	2237	182	4	8.31%
		+CNN	2241	374	39	18.43%
		+UNet	2256	725	866	70.52%
100	3929	/	2227	101	3	4.67%
		+CNN	2231	369	52	18.87%
		+UNet	2239	704	887	**71.06%**
200	3469	/	2056	50	1	2.48%
		+CNN	2058	268	31	14.53%
		+UNet	2060	651	750	68.01%
500	2819	/	1750	19	0	1.09%
		+CNN	1751	139	9	8.45%
		+UNet	1751	635	316	54.31%
1000	2391	/	1578	13	0	0.82%
		+CNN	1580	74	1	4.75%
		+UNet	1581	443	77	32.89%

We used a screening window of 400 bp length for refining breakpoints predicted by CNVnator using different bin sizes. Candidate breakpoints were placed in the center of the screening window in the enhancement step, which is different from that in the training step. For the candidate breakpoints predicted by CNVnator using bin size no greater than 200 bp, at least one full-length bin on each side of a breakpoint is overlapped by the screening window. For those breakpoints predicted by CNVnator using bin size greater than 200 bp, the screening window may only cover a partial length of a bin. The gold-standard breakpoint contained by the bin may be outside of the screening window for such a partial coverage case. This observation explains that the number of enhanced SVs with precise boundaries reduces significantly for CNVnator using bin size greater than 200 bp. Empirically, the screening window covering at least four-bin length is preferred (two bins on either side).

We investigated the number of changed SVs with precise breakpoints before and after the enhancement. From Table 2, we can observe: First, the number of SVs with precise boundaries increases significantly. Before enhancement, due to the bin-size limitation, SVs with precise boundaries are very few. For example, CNVnator predicted up to four both-boundary-match SVs with the bin size of 50 bp. The number of partial-boundary-match SVs is also less than 10% of the predicted gold-standard overlapping SVs. After enhancement, the number of both-boundary-match SVs increases up to 887. For CNVnator using the bin size of 50 bp, the proportion of SVs with precise boundaries increases from 8.31% to 70.52% with the UNet enhancement. For the enhancement using CNN, the number of SVs with precise boundaries increases to 18.43%. These demonstrate the effectiveness and the potential of using UNet for enhancing RD-based SV callers. Second, the number of gold-standard overlapping SVs (JS > 0.5) slightly increases. Compared with the predicted SVs by CNVnator using the bin size of 50 bp, there are 4 and 19 more gold-standard overlapping SVs after the enhancement using CNN and UNet, respectively. These changes are due to the adjustment of breakpoints, which makes more SVs with Jaccard similarity greater than 0.5. Third, UNet also achieves a better performance than CNN in that there are more partial-boundary-match SVs and significantly more both-boundary-match SVs using the UNet enhancement.

**Fig 3:**
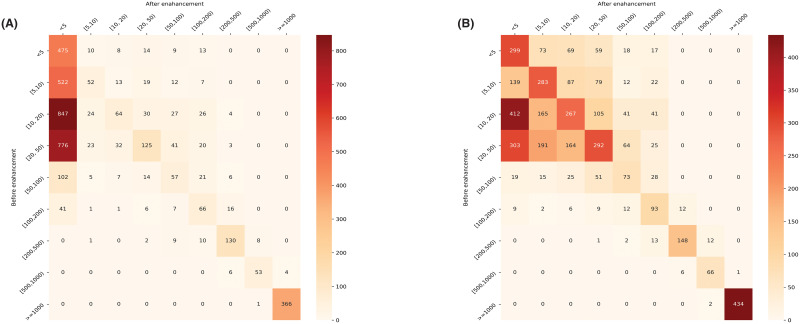
Change matrices for evaluating the enhancement effect of the UNet and CNN model. (A) Change matrix of the enhancement using the UNet model. (B) Change matrix of the enhancement using the CNN model.

We further investigated the enhancement effect on breakpoints. For each breakpoint, we evaluated the change of its distance to the gold standard (to-GS-distance). We counted the number of breakpoints in different to-GS-distance ranges. Fig 3 shows the number of breakpoint changes before and after the enhancement using UNet and CNN. We demonstrate changes in the form of a confusion matrix plotted in a heatmap. We split to-GS-distance into a range set of DR={[0,5), [5,10), [10,20), [20,50), [50,100), [100,200), [200, 500), [500, 1000), [1000,)}. For each element in the change matrix, *c*_*i*,*j*_ represents the number of breakpoints changing from *DR*_*i*_ to *DR*_*j*_ after enhancement. For *c*_*i*,*i*_ that represents breakpoints adjusted in the same range, we ignored those unchanged breakpoints. For visualization, matrix elements with larger values are plotted in darker colors. UNet enhances 2145 breakpoints with their original to-GS-distance in the range between 5 bp to 50 bp to the breakpoints with to-GS-distance less than 5 bp, as shown in Fig 3A. The number of positive enhancements of CNN is less than that of UNet. Note that there are negative adjustments that make to-GS-distance increasing. The number is smaller than the number of positive enhancements. We took error analysis and found that several negative adjustments belong to the case that gold-standard breakpoints are too far away from the initially predicted coordinates, which are also out of the screening window.

#### Comparisons of enhanced RD-based SV callers and other types of SV callers

**Fig 4:**
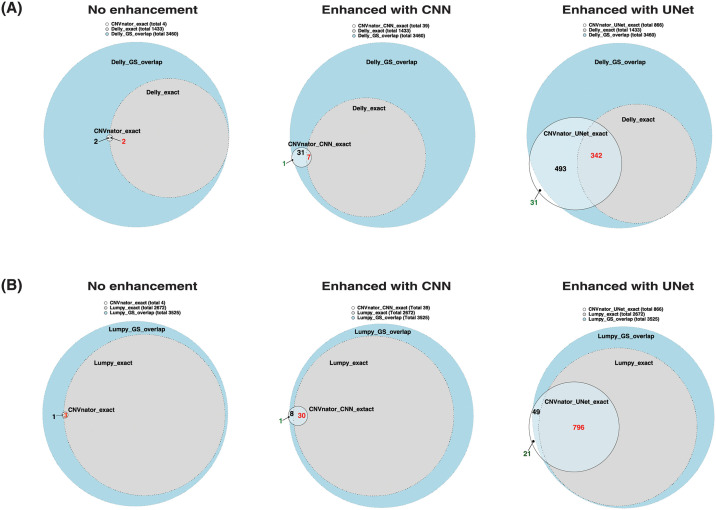
Comparison of the enhanced RD-based SV callers with two different SV callers on the simulated data. Evaluated SVs are the ones that overlap with the gold-standard SVs (Jaccard similarity > 0.5). SVs with exact breakpoints (both-boundary-match, which is abbreviated as “*_exact”) predicted by CNVnator (w/wo enhancement) are compared with the GS-ov SVs predicted by Delly and Lumpy, which are shown in the following Venn diagrams. Any two predicted SVs are treated as overlapped as long as they overlap with the same gold-standard SV. The Venn diagrams were plotted using Eulerr [20]. (A) Comparison of CNVnator (w/wo enhancement) and Delly. (B) Comparison of CNVnator (w/wo enhancement) and Lumpy.

As existing RD-based callers are not designed to predict breakpoints in single-nucleotide resolution, we compared the proposed method with other types of SV callers that can make predictions in single-nucleotide resolution. Here, we used Delly (v 0.8.1) [7] and Lumpy (v 0.2.13) [19]. Delly detects SVs based on abnormally aligned reads, including discordant-paired-end reads and split reads. Lumpy integrates paired-end aligner, split-read aligner, and CNV prediction under a probabilistic framework for SV discovery. On the simulated data, Delly predicted a total of 3460 gold-standard overlapping SVs (Jaccard similarity > 0.5, 1433 SVs (41.4%) are both-boundary-match), while Lumpy predicted a total of 3525 gold-standard overlapping SVs (2672 SVs (75.8%) are both-boundary-match). These numbers of gold-standard overlapping SVs are around 1.5 times more than the CNVnator using the bin size of 50 bp (total 2237 SVs) on the same data. Around 90.82% and 92.95% of gold-standard overlapping SVs predicted by the CNVnator are included in the predictions of Delly and Lumpy, respectively. Limited by the bin size of 50 bp, the predicted SVs with both-boundary-match by the non-enhanced CNVnator are very few (only four SVs). We then enhanced CNVnator with different segmentation models of CNN and UNet. We compared the SVs with both-boundary-match predicted by enhanced CNVnators with the GS-ov SVs predicted by Delly and Lumpy, as shown in Fig 4. The UNet-based enhancement significantly increases the number of both-boundary-match SVs to 866. These 866 SVs have 39.5% and 91.9% of both-boundary-match SVs that are overlapped by those predicted by Delly and Lumpy, respectively. There are 31 and 21 GS-ov SVs that are not overlapped with any GS-ov SVs predicted by Delly and Lumpy. The number of both-boundary-match SVs (total 39) with the CNN enhancement is much less than that of the UNet enhancement (total 866). There is only one SV that is not overlapped with any GS-ov SVs predicted by Delly and Lumpy.

### Real data performance

We evaluated model-level performance on the two benchmark samples, NA12878 and HG002. In Table 3, we observe a similar tendency as in the evaluation of the simulated data. UNet gives better segmentation performance of higher DSC scores than that of CNN, which are around 2% ∼ 2.36% higher on NA12878, and 2.43% ∼ 3.88% higher on HG002 with different amounts of training data. UNet also achieves the best performance in all evaluation metrics in the classification task. Meanwhile, only using 20% of training data, the performance of UNet does not decrease as significantly as that of CNN. In the classification task, FDR values of the three models on NA12878 are higher than those on the simulated data, although NA12878 and the simulated data have SVs in common. One reason is related to the precision of breakpoints of the dataset. For the real dataset, breakpoints are integrated from several SV callers and defined in a confidence interval that ranges from zero bp to several hundred bp. While in the simulated data, breakpoints are more precisely defined. This observation indicates that more accurate data is essential for training a segmentation model to achieve a lower FDR value.

**Table 3:** Model-level performance on the real dataset. 5-fold cross-validations were performed on NA12878 and HG002 using a screening window length of 400 bp. The average result of 5-repeat runs is reported to reduce the effect of the randomness of GPU training.

		NA12878						HG002					
Model	Train-data proportion	Segmentation		Classification				Segmentation		Classification			
		DSC-ALL	DSC-BK	F1 score	Precision	Recall	FDR	DSC-ALL	DSC-BK	F1 score	Precision	Recall	FDR
SVM	80%	/		0.8371	0.8403	0.834	0.1597	/		0.8808	0.891	0.8709	0.109
	20%	/		0.8323	0.8351	0.8296	0.1649	/		0.8747	0.8885	0.8614	0.1115
CNN	80%	0.7688	0.8066	0.8546	0.8595	0.8505	0.1405	0.8024	0.8277	0.8956	0.8880	0.9035	0.1120
	20%	0.7466	0.7862	0.8364	0.8575	0.8174	0.1425	0.7719	0.7987	0.8857	0.8842	0.8880	0.1158
UNet	80%	0.7911	0.8266	0.8698	0.8817	0.8595	0.1183	0.8284	0.8520	0.9117	0.9163	0.9076	0.0837
	20%	0.7696	0.8098	0.8587	0.8709	0.8480	0.1291	0.8107	0.8358	0.9021	0.9087	0.8963	0.0913

#### In-sample enhancement on real data

We conducted in-sample enhancement using four real samples. We randomly selected 20% of known SVs and generated RD vectors surrounding SV boundaries to train UNet and CNN on each sample. The rest 80% of SVs were used for evaluating the enhancement effect of the CNVnator using 50 bp bin size. Table 4 shows the number of changes of specific SVs and breakpoints before and after the enhancement. UNet predicted more SVs with precise boundaries (“l/r match” and “l&r match”) than CNN for all four samples. For HG002, the total percentage of partial-boundary-match SVs and both-boundary-match SVs is around 59.72%. Besides, we observe that the number of breakpoints with the to-GS-distance less than 5 bp also increases significantly after the UNet enhancement. The mean of to-GS-distances less than 5 bp also reduces, as shown in S4 Table. The change-matrices are shown in S1 Fig. Most of the positively enhanced breakpoints by the UNet model are these with the original to-GS-distance in the range between 20 bp and 100 bp. While for the CNN model, its enhancement effect is relatively conservative in that fewer breakpoints are enhanced with the to-GS-distance less than 5 bp.

**Table 4:** In-sample enhancement for the CNVnator using 50 bp bin size. For each SV caller, the largest number of breakpoints in the split regions is highlighted in bold. “GS-ov” SVs are referred to as gold-standard overlapping SVs. “l/r match” represents SVs with partial-boundary-match (left or right). “l&r match” denotes SVs with both-boundary-match (left and right).

CNVnator			Specific SVs w/wo enhancement				Breakpoints in different to-GS-distance (bp) ranges								
Sample	predicted SVs	enhancement	GS-ov SVs	(1) l/r match	(2) l&r match	$\frac{\left(1)+(2\right)}{\text{GS-ov}\;\text{SVs}}$	<5	[5, 10)	[10 20)	[20, 50)	[50, 100)	[100, 200)	[200, 500)	[500, 1k)	[1k,)
NA12878	8649	/	598	8	0	1.34%	33	46	98	**461**	339	75	76	28	40
		+CNN	600	23	1	4.0%	66	78	215	**423**	172	106	71	28	41
		+UNet	599	121	53	29.0%	**373**	100	163	174	133	113	73	29	40
NA19238	10049	/	635	7	0	1.1%	30	37	90	**449**	410	99	57	47	51
		+CNN	636	40	1	6.45%	156	131	220	**314**	179	110	66	48	48
		+UNet	637	139	61	31.4%	**397**	104	126	183	166	127	73	46	52
NA19239	10747	/	683	9	0	1.32%	29	37	61	**523**	442	110	67	43	54
		+CNN	686	29	0	4.23%	95	104	254	**474**	173	106	71	39	56
		+UNet	686	169	79	36.15%	**478**	115	137	179	144	145	81	41	52
HG002	15244	/	1187	27	0	2.27%	69	84	302	**1116**	521	125	88	40	29
		+CNN	1195	117	6	10.25%	347	375	520	**609**	263	118	89	42	27
		+UNet	1199	569	147	59.72%	**1159**	198	270	252	197	146	106	44	26

#### Cross-sample enhancement on real data

We then evaluated the segmentation models across different samples. The cross-sample evaluation investigated whether the models can be generalized across samples. We assumed samples are sequenced on the same platform. Here, we used WGS data from the 1kGP for satisfying this pre-condition. We used the comprehensively studied sample NA12878 to train deep segmentation models and applied the trained models for NA19238 and NA19239. Different from the in-sample evaluation that 20% of SVs used in training were excluded, all known SVs of the target sample were evaluated.

Table 5 shows the cross-sample evaluation at the model and application level. At the model level, recalls of all the models are relatively lower, while precision scores remain when comparing with the in-sample evaluation. UNet achieves the best segmentation and classification performance. SVM has the largest FDR value of 0.2045 and 0.1626 on NA19238 and NA19239, respectively. This result indicates that classification based on segmentation results works better than the classification making direct predictions for the cross-sample application. At the application level, a similar enhancement effect as in the in-sample evaluation can be observed for the UNet model. The proportion of SVs with precise boundaries increases from around 1.3% to 34.39% and 36.26%. The mean and standard derivation of to-GS-distances in different ranges are shown in S5 Table. The performance of CNN has a noticeable improvement when compared with the in-sample case. Breakpoint change matrices of the samples are shown in S2 Fig and more breakpoints are enhanced with the to-GS-distance less than 5 bp. As discussed in the simulation study, CNN is more sensitive to the amount of training data. In the cross-sample evaluation, more data were used for training, which contributes to the performance improvement of CNN. However, the number of SVs with precise boundaries and the number of breakpoints with the to-GS-distance less than 5 bp are still less than that of UNet.

**Table 5:** Cross-sample enhancement on the real data. Deep segmentation models were trained on NA12878, and enhancements using different models were applied for NA19238 and NA19239. “GS-ov SVs” is referred to as gold-standard overlapping SVs. “l/r match” represents SVs with partial-boundary-match (left or right), and “l&r match” represents SVs with both-boundary-match (left and right).

Sample	Model	**Segmentation**		**Classification**			
		DSC-ALL	DSC-BK	F1 score	Precision	Recall	FDR
NA19238	SVM	/		0.7095	0.7955	0.6403	0.2045
	CNN	0.6725	0.7020	0.7922	0.8856	0.7167	0.1145
	UNet	0.7132	0.7335	0.8008	0.9373	0.6990	0.0627
NA19239	SVM	/		0.6972	0.8374	0.5972	0.1626
	CNN	0.6222	0.6498	0.7396	0.8783	0.6388	0.1217
	UNet	0.6929	0.7263	0.7542	0.8918	0.6534	0.1082

CNVnator			Specific SVs w/wo enhancement				Breakpoints in different to-GS-distance (bp) ranges								
Sample	predicted	enhanced	GS-ov SVs	(1) l/r match	(2) l&r match	$\frac{\left(1)+(2\right)}{\text{GS-ov}\;\text{SVs}}$	<5	[5, 10)	[10 20)	[20, 50)	[50, 100)	[100, 200)	[200, 500)	[500, 1k)	[1k,)
NA19238	10049	/	786	10	0	1.27%	34	44	100	**561**	514	116	80	58	65
		+CNN	786	120	15	17.18%	**389**	222	214	244	172	121	93	59	58
		+UNet	788	201	70	34.39%	**556**	125	154	216	196	115	89	63	62
NA19239	10747	/	837	11	0	1.3%	36	44	82	**623**	562	124	86	56	61
		+CNN	841	121	7	15.22%	**397**	266	192	271	212	136	97	51	60
		+UNet	841	227	78	36.26%	**603**	115	154	258	205	135	102	49	61

#### Cross-sample enhancement on tumor WGS data

We performed an additional investigation on UNet’s generalization ability on tumor WGS data. We used WGS data of COLO829 tumor cell line provided by Valle-Inclan et al. [21]. SVs were validated with orthogonal technologies, including Illumina Hiseq, Oxford nanopore, Pacific biosciences, and 10x genomics for the sample. Besides that, additional validations, such as capture probe, PCR, and Bionano, were also applied. We used the provided alignment (BAM file, around 100x coverage) and the SV truthset that contains 32 DELs and 7 DUPs on autosomes (Insertions and translocations were excluded from the evaluation). We trained the UNet model on NA12878 data and tested it on the COLO829 tumor WGS dataset (without using paired normal WGS data). We used the default setting as in the previous experiments that the length of the screening window is 400 bp, and the bin size of the CNVnator is 50 bp. The change matrix of the UNet enhancement is shown in S3 Fig. From the figure, we observe that four breakpoints are enhanced with a higher resolution within the 5 bp range, and two breakpoints are enhanced to the range of [5, 10]. This result demonstrates a generalization ability of the UNet model on the tumor WGS data.

### Effect of read depth and screening window length

**Fig 5:**
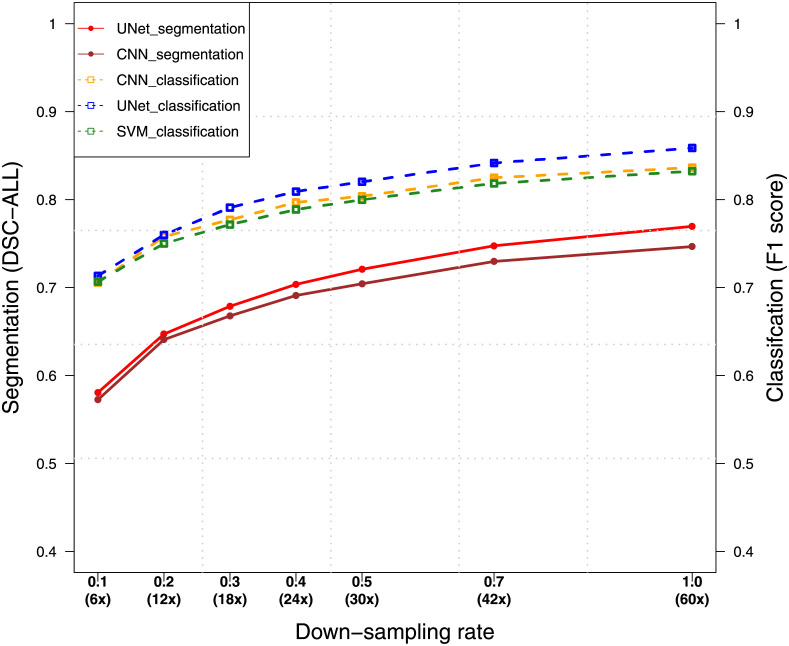
Performance of models using data of different read depths on the in-sample evaluation. Different read-depth data were generated through down-sampling NA12878 WGS data of 60x read depth. The dashed curves connect F1 scores of classification, while the line curves show DSC-ALL scores of segmentation.

The breakpoint enhancement is affected by read depth. We empirically evaluated how read depth affects model performance through down-sampling the WGS data of NA12878. We conducted 5-fold cross-validation with 20%-train-split for NA12878 of different read depths. As shown in Fig 5, we observe a general trend of performance improvement as the increase of read depth. Curves in the region of the down-sampling rate below 0.5 are relatively steeper compared with the curves in the rest regions. The figure indicates the difficulty of training a deep segmentation model for a single sample using low-depth data. Based on performance gaps shown in the figure, read depth no less than 40x is empirically suggested for applying the UNet model.

**Fig 6:**
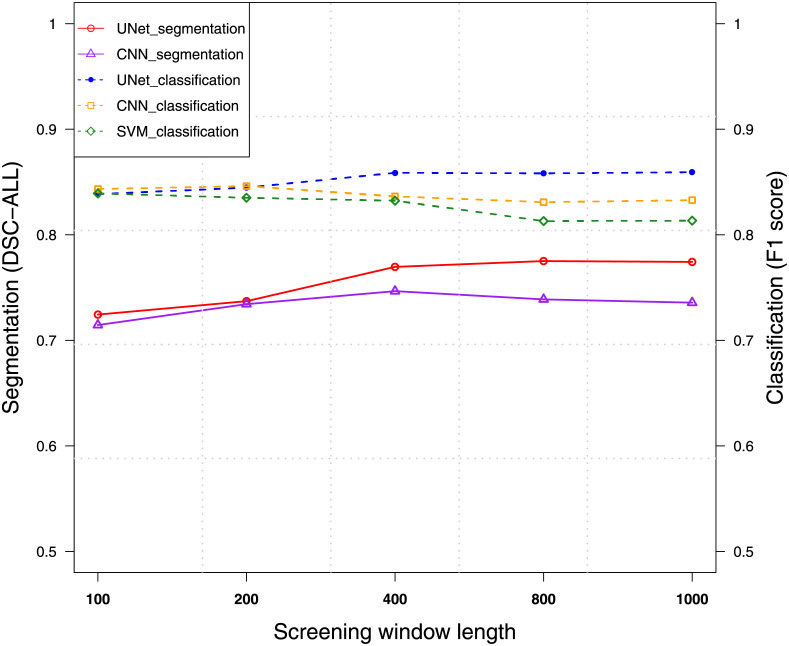
Performance of models using different screening window lengths on NA12878 WGS data of 60x read depth. The dashed curves connect F1 scores of classification, while the line curves show DSC-ALL scores of segmentation.

To evaluate the effect of the screening window length, we used NA12878 to perform in-sample enhancement for the CNVnator using 50 bp bin size. The screening window length of 100 bp, 200 bp, 400 bp, 800 bp, and 1000 bp were evaluated. As shown in Fig 6, at the model level, different screening window length does not make drastic performance changes as were affected by the read-depth. For the classification task, we observe the performances of SVM and CNN slightly decrease as the length of the screening window increases, while the F1 curve of UNet starts to diverge from that of SVM and CNN after the length of 200 bp. Meanwhile, it has a trend of converging with higher F1 scores than that of SVM and CNN. The longer the screening window is, the more RDs are included. SVM and CNN are more likely to be affected by using more base-wise RD signals, while UNet generalizes well for the screen window with longer lengths. As the screening window length increases for the segmentation task, the number of evaluated labels also increases. The DSC scores increase accordingly. UNet performs better than CNN, especially with the screening window length greater than 400 bp.

## Discussion

In this paper, we proposed RDBKE, a general enhancement approach to increase the breakpoint resolution for read-depth based SV callers. The core component of RDBKE uses the UNet model to segment regions surrounding candidate breakpoints. Previous RD-based SV callers usually require smoothed RD signals by bins, limiting the resolution of predicted breakpoints. Here, we used UNet to process base-wise RD signals directly and refined breakpoint predictions in single-nucleotide resolution. Although the base-wise RD of WGS data is very noisy, a deep learning model with a proper neural network structure can be used to process it directly. Besides the convolutional module, the encoding-decoding architecture and the skip-connection structure contribute to this functionality. Furthermore, the UNet model can be trained with a small amount of data, making the in-sample application practically feasible. The enhancement pipeline can also be applied for the cross-sample application using more training data under the condition that both samples are sequenced on the same platform.

Usually, an SV callset is generated through clustering predictions of different SV callers. For those benchmark SV callsets, multiple sequencing technologies are also applied for the same sample to derive high-confidence results. Several computational algorithms [22, 23] have been developed for further filtering and merging SV predictions. For example, SVclassify [23] used one-class SVM to cluster and classify whether candidate SVs have abnormal annotations different from most of the genome. Here, we focused on the RD-based SV caller. Instead of developing a new RD-based SV caller, we proposed using deep learning models to enhance existing RD-based SV callers. Related but different from existing machine learning applications for SV detection that are modeled as classification tasks, we took a different modeling approach as the segmentation task, which can provide a better granularity for the analysis of putative regions.

Compared with other types of information, the regular RD is more common in the whole-genome scale. Although the split-read based methods can accurately predict base-wise breakpoints, there is a limited number of SVs that are overlapped by plenty of split-reads [24]. Pedersen et al., [25] proposed to integrate read-depth information to analyze putative events generated through the clustering of discordant-read and split-read based algorithms. The method compared the median depth in the event to the median depth from the 1000 bases on either side, which is used to refine predictions of split-read and paired-end based methods. However, this method still used bin-based RDs.

We took error analysis on the output of the segmentation models on the simulated data. For those regions containing drastic RD changes, it is relatively easy for UNet and CNN to label SV-overlapping coordinates. UNet tends to predict more consecutive identical label marks than CNN, especially near candidate boundaries. For the regions with less drastic RD change, it is still possible for both deep segmentation models to make almost correct segments, as shown in Table A in S4 Fig. Both segmentation models show cases that detect small size SVs (Table B in S4 Fig). Besides the issue of gold-standard breakpoints outside the screening window, the other two types of errors are observed. One is that the RD signal is less informative to make reliable segmentation. The other is the inconsistency of breakpoint annotations that some adjacent known breakpoints have different RD signal patterns, as shown in Table C in S4 Fig. Although the segmentation is not perfect, our experiments demonstrate that the base-wise RDs can still be used to learn specific signal patterns surrounding breakpoints for refinement. We expect the performance can be further improved when high-coverage and high-quality training data become more and more available. To alleviate the effect of wrong segments, the original SVs can also be retained along with the enhancement result for further clustering-based analysis.

In this work, we only used the read-depth information to enhance the breakpoint resolution of SVs. The proposed deep learning framework also has the flexibility of incorporating other different features as the input. For example, we can incorporate sequence-related information and read-depth of specific reads, such as split-reads and paired-end reads. DeepVariant used pileup images of putative regions (100 bp) and applied CNN to classify genotypes for detecting SNPs and InDels. Although the pileup image representation contains more information than one-dimensional RD representation, they are much noisier, especially when putative regions are usually larger in the SV detection task. On the other hand, this method can also be extended for long-read WGS data through learning RD patterns of long-reads. It is worth exploring further.

## Conclusion

In this paper, we presented RDBKE for enhancing breakpoints in single-nucleotide resolution for RD-based SV callers. RDBKE used UNet to learn base-wise RD patterns surrounding known breakpoints. We showed that UNet can be trained with a small amount of data and can be applied for breakpoint enhancement in-sample and cross-sample. RDBKE using UNet can significantly increase SVs’ number with more precise breakpoints on both simulated and real data.

## Supporting information

S1 Table Data source of related VCF and BAM files of the simulated and real data. (A) Data source of VCF files. (B) Data source of BAM files.
(PDF)

S2 Table Detailed network structures of UNet and CNN. (A) UNet network structure. (B) CNN network structure.
(PDF)

S3 Table Results of repeat runs of the 5-fold cross-validation on the simulated data. (XLSX)

S4 Table Mean and standard derivation of to-GS-distances of breakpoints in different ranges for the in-sample evaluation on the real data. (XLSX)

S5 Table Mean and standard derivation of to-GS-distances of breakpoints in different ranges for the cross-sample evaluation on the real data. (XLSX)

S1 Fig Breakpoint change matrices of the in-sample enhancement on NA12878, NA19238, NA19239, and HG002 WGS data. For NA12878 WGS data, (A), (C), (E), and (G) are the results of enhancement using UNet for NA12878, NA19238, NA19239, and HG002, respectively. (B), (D), (F), and (H) are the results of enhancement using CNN for NA12878, NA19238, NA19239, and HG002, respectively.
(DOCX)

S2 Fig Breakpoint change matrices of cross-sample enhancement on NA19238, NA19239 WGS data. For NA19238 WGS data, (A) and (B) are the results of enhancement using UNet and CNN, respectively. For NA19239 WGS data, (C) and (D) are the results of enhancement using UNet and CNN, respectively.
(DOCX)

S3 Fig Breakpoint change matrix of the UNet enhancement on COLO829 tumor WGS data. (DOCX)

S4 Fig Segmentation results of specific cases on the simulated data. The dashed line indicates the position of the gold-standard breakpoint. Coordinates in red are the SV-overlapping coordinates. (A) Cases of positive enhancement by UNet. (B) Cases of small SVs entirely inside the screening window. (C) Negative segmentation examples of UNet.
(DOCX)
