# Supplementary material for: Enhancing breakpoint resolution with deep segmentation model: A general refinement method for read-depth based structural variant callers
Source: PLoS Comput Biol. 2021 Oct 11;17(10):e1009186. doi: 10.1371/journal.pcbi.1009186 (PMC8504719; doi:10.1371/journal.pcbi.1009186)
Supplement: S1 Table — (A) Data source of VCF files. (B) Data source of BAM files. (PDF) [file pcbi.1009186.s001.pdf]

S1 Table. Data source of related VCF and BAM files of the simulated and real data.

(A) Data source of VCF files

Samples	VCF files
Simulation	https://github.com/stat-lab/EvalSVcallers/blob/master/Ref_SV/Sim-A.SV.vcf
NA12878, NA19238, NA19239	ftp://ftp.1000genomes.ebi.ac.uk/vol1/ftp/phase3/integrated_sv_map/ALL.wgs.mergedSV.v8.20130502.svs.genotypes.vcf.gz
HG002	ftp://ftp-trace.ncbi.nlm.nih.gov/giab/ftp/data/AshkenazimTrio/analysis/NIST_SVs_Integration_v0.6/HG002_SVs_Tier1_v0.6.vcf.gz
COLO829T	https://zenodo.org/record/3988185/files/truthset_somaticSVs_COLO829.vcf?download=1

(B) Data source of BAM files

Samples	BAM files
NA12878	ftp://ftp.1000genomes.ebi.ac.uk/vol1/ftp/data/NA12878/high_coverage_alignment/NA12878.mapped.ILLUMINA.bwa.CEU.high_coverage_pcr_free.20130906.bam
NA19238	ftp://ftp.1000genomes.ebi.ac.uk/vol1/ftp/data/NA12878/high_coverage_alignment/NA19238.mapped.ILLUMINA.bwa.YRI.high_coverage_pcr_free.20130924.bam
NA19239	ftp://ftp.1000genomes.ebi.ac.uk/vol1/ftp/data/NA12878/high_coverage_alignment/NA19239.mapped.ILLUMINA.bwa.YRI.high_coverage_pcr_free.20130924.bam
HG002	ftp://ftp-trace.ncbi.nlm.nih.gov/giab/ftp/data/AshkenazimTrio/HG002_NA24385_son/NIST_HiSeq_HG002_Homogeneity-10953946/NHGRI_Illumina300X_AJtrio_novoalign_bams/HG002.hs37d5.60X.1.bam.
COLO829T	ftp://ftp.sra.ebi.ac.uk/vol1/run/ERR275/ERR2752450/COLO829T_dedup.realigned.bam
