# Supplementary material for: Enhancing breakpoint resolution with deep segmentation model: A general refinement method for read-depth based structural variant callers
Source: PLoS Comput Biol. 2021 Oct 11;17(10):e1009186. doi: 10.1371/journal.pcbi.1009186 (PMC8504719; doi:10.1371/journal.pcbi.1009186)
Supplement: S2 Table — (A) UNet network structure. (B) CNN network structure. (PDF) [file pcbi.1009186.s002.pdf]

S2 Table. Detailed network structures of UNet and CNN.

(A) UNet network structure.

Layer (type)	Output Shape	Param #	Connected to
input (InputLayer)	(None, 400, 1)	0	
conv1d_1 (Conv1D)	(None, 400, 32)	384	input[0][0]
batch_normalization_1 (BatchNor	(None, 400, 32)	128	conv1d_1[0][0]
conv1d_2 (Conv1D)	(None, 400, 32)	11296	batch_normalization_1[0][0]
batch_normalization_2 (BatchNor	(None, 400, 32)	128	conv1d_2[0][0]
max_pooling1d_1 (MaxPooling1D)	(None, 80, 32)	0	batch_normalization_2[0][0]
conv1d_3 (Conv1D)	(None, 80, 64)	22592	max_pooling1d_1[0][0]
batch_normalization_3 (BatchNor	(None, 80, 64)	256	conv1d_3[0][0]
conv1d_4 (Conv1D)	(None, 80, 64)	45120	batch_normalization_3[0][0]
batch_normalization_4 (BatchNor	(None, 80, 64)	256	conv1d_4[0][0]
max_pooling1d_2 (MaxPooling1D)	(None, 16, 64)	0	batch_normalization_4[0][0]
conv1d_5 (Conv1D)	(None, 16, 128)	90240	max_pooling1d_2[0][0]
batch_normalization_5 (BatchNor	(None, 16, 128)	512	conv1d_5[0][0]
conv1d_6 (Conv1D)	(None, 16, 128)	180352	batch_normalization_5[0][0]
batch_normalization_6 (BatchNor	(None, 16, 128)	512	conv1d_6[0][0]
max_pooling1d_3 (MaxPooling1D)	(None, 8, 128)	0	batch_normalization_6[0][0]
conv1d_7 (Conv1D)	(None, 8, 256)	360704	max_pooling1d_3[0][0]
batch_normalization_7 (BatchNor	(None, 8, 256)	1024	conv1d_7[0][0]
conv1d_8 (Conv1D)	(None, 8, 256)	721152	batch_normalization_7[0][0]
batch_normalization_8 (BatchNor	(None, 8, 256)	1024	conv1d_8[0][0]
up_sampling1d_1 (UpSampling1D)	(None, 16, 256)	0	batch_normalization_8[0][0]
concatenate_1 (Concatenate)	(None, 16, 384)	0	batch_normalization_6[0][0]<br>up_sampling1d_1[0][0]
conv1d_9 (Conv1D)	(None, 16, 128)	540800	concatenate_1[0][0]
batch_normalization_9 (BatchNor	(None, 16, 128)	512	conv1d_9[0][0]
conv1d_10 (Conv1D)	(None, 16, 128)	180352	batch_normalization_9[0][0]
batch_normalization_10 (BatchNo	(None, 16, 128)	512	conv1d_10[0][0]
up_sampling1d_2 (UpSampling1D)	(None, 80, 128)	0	batch_normalization_10[0][0]
concatenate_2 (Concatenate)	(None, 80, 192)	0	batch_normalization_4[0][0]<br>up_sampling1d_2[0][0]
conv1d_11 (Conv1D)	(None, 80, 64)	135232	concatenate_2[0][0]
batch_normalization_11 (BatchNo	(None, 80, 64)	256	conv1d_11[0][0]
conv1d_12 (Conv1D)	(None, 80, 64)	45120	batch_normalization_11[0][0]
batch_normalization_12 (BatchNo	(None, 80, 64)	256	conv1d_12[0][0]
up_sampling1d_3 (UpSampling1D)	(None, 400, 64)	0	batch_normalization_12[0][0]
concatenate_3 (Concatenate)	(None, 400, 96)	0	batch_normalization_2[0][0]<br>up_sampling1d_3[0][0]
conv1d_13 (Conv1D)	(None, 400, 32)	33824	concatenate_3[0][0]
batch_normalization_13 (BatchNo	(None, 400, 32)	128	conv1d_13[0][0]
conv1d_14 (Conv1D)	(None, 400, 32)	11296	batch_normalization_13[0][0]
batch_normalization_14 (BatchNo	(None, 400, 32)	128	conv1d_14[0][0]
conv1d_15 (Conv1D)	(None, 400, 2)	706	batch_normalization_14[0][0]
batch_normalization_15 (BatchNo	(None, 400, 2)	8	conv1d_15[0][0]
conv1d_16 (Conv1D)	(None, 400, 1)	3	batch_normalization_15[0][0]

Total params: 2,384,813
Trainable params: 2,381,993
Non-trainable params: 2,820

Train params used:
{"lr": 0.001, "batchSize": 64, "conv_window_len": 11, "stride":1, "kernel_size":[32, 64, 128, 256], "maxpooling_len": [5, 5, 2], "epoch": 100, "DropoutRate": 0}

(B) CNN network structure

Layer (type)	Output Shape	Param #
conv1d_1 (Conv1D)	(None, 390, 64)	768
max_pooling1d_1 (MaxPooling1	(None, 78, 64)	0
conv1d_2 (Conv1D)	(None, 72, 128)	57472
max_pooling1d_2 (MaxPooling1	(None, 14, 128)	0
flatten_1 (Flatten)	(None, 1792)	0
dense_1 (Dense)	(None, 256)	459008
dropout_1 (Dropout)	(None, 256)	0
dense_2 (Dense)	(None, 400)	102800

Total params: 620,048
Trainable params: 620,048
Non-trainable params: 0

Train params used:
{"lr": 0.001, "batchSize": 64, , "kernel_size":[64,128], "window_len": [11,7], "maxpooling_len": [5, 5], "epoch": 100, "DropoutRate": 0.2, "BN": false}
