# Supplementary material for: Enhancing breakpoint resolution with deep segmentation model: A general refinement method for read-depth based structural variant callers
Source: PLoS Comput Biol. 2021 Oct 11;17(10):e1009186. doi: 10.1371/journal.pcbi.1009186 (PMC8504719; doi:10.1371/journal.pcbi.1009186)

**S1 Fig. Breakpoint change matrices of the in-sample enhancement on NA12878, NA19238, NA19239, and HG002 WGS data. For NA12878 WGS data, (A), (C), (E), and (G) are the results of enhancement using UNet for NA12878, NA19238, NA19239, and HG002, respectively. (B), (D), (F), and (H) are the results of enhancement using CNN for NA12878, NA19238, NA19239, and HG002, respectively.**

**NA12878**

1. **Enhancement using UNet on NA12878 (B) Enhancement using CNN on NA12878**


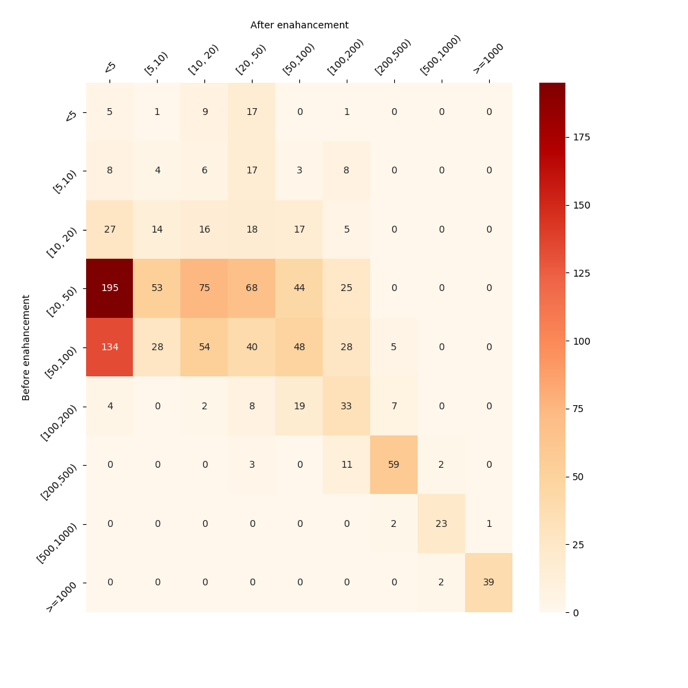

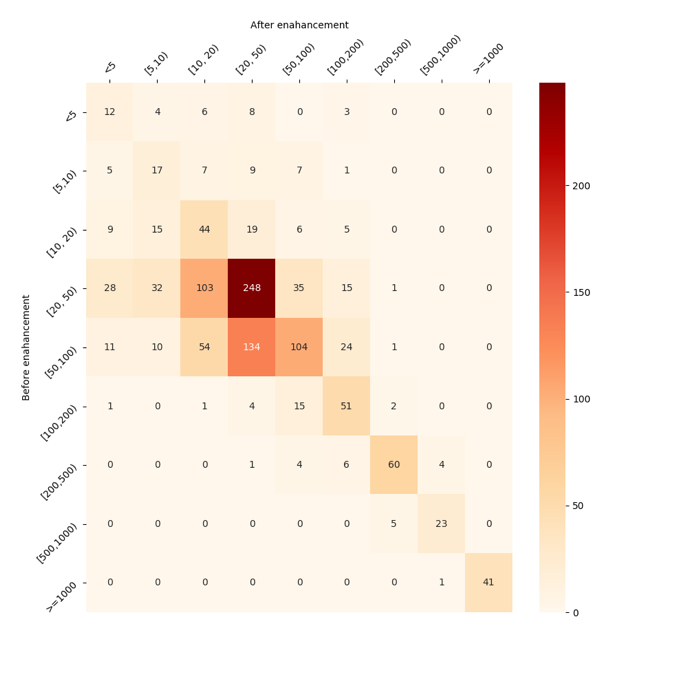


**NA19238**

**(C) Enhancement using UNet on NA19238 (D) Enhancement using CNN on NA19238**


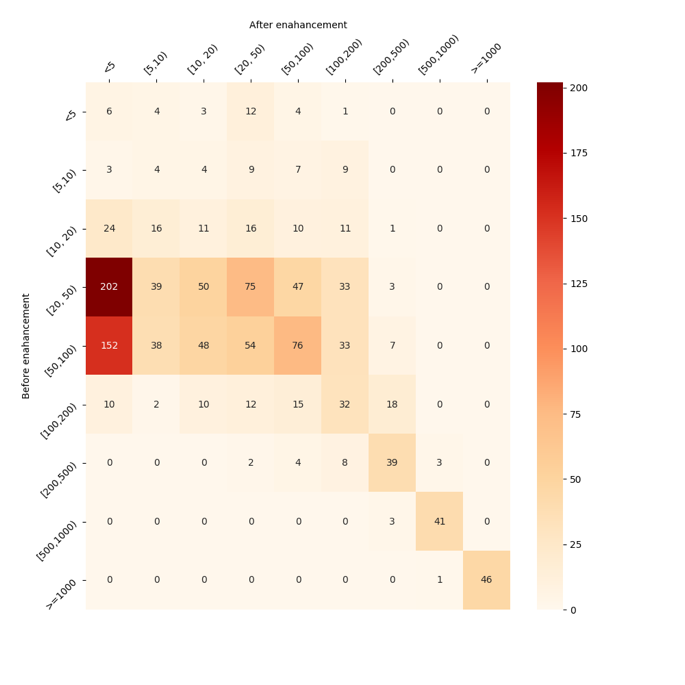

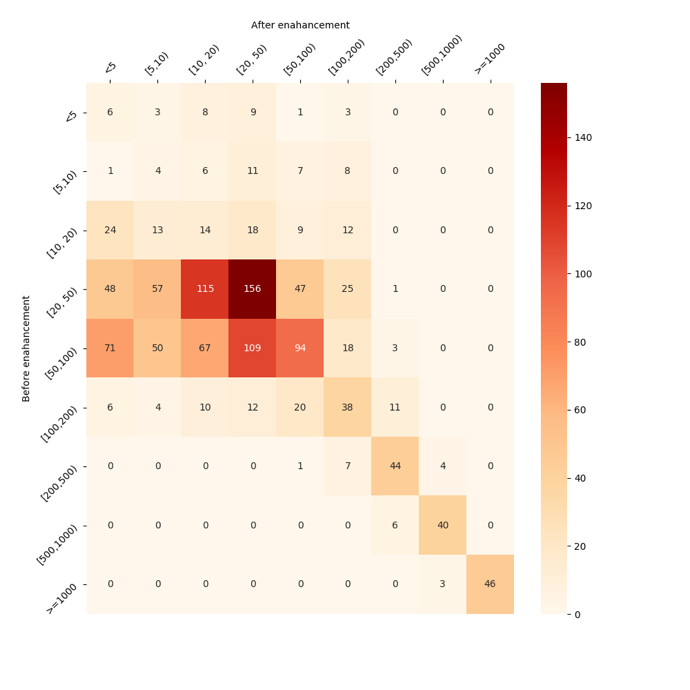


**NA19239**

**(E) Enhancement using UNet on NA19239 (F) Enhancement using CNN on NA19239**


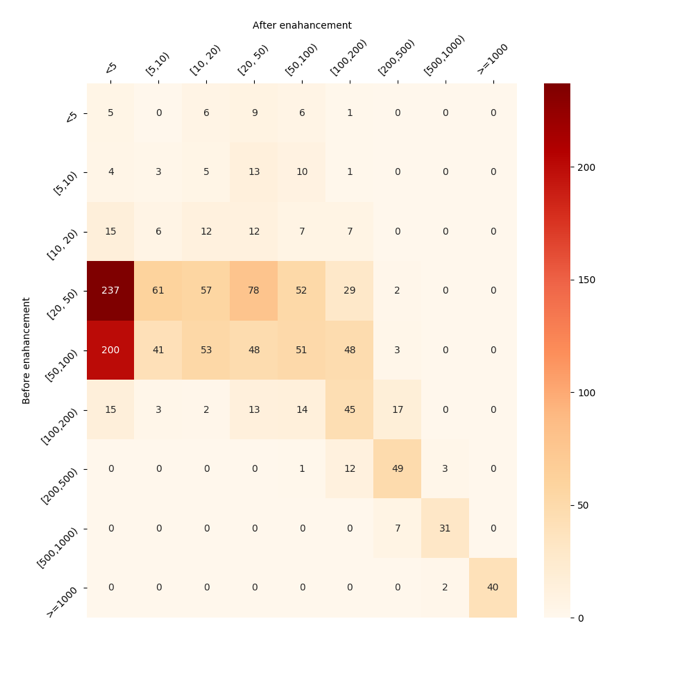

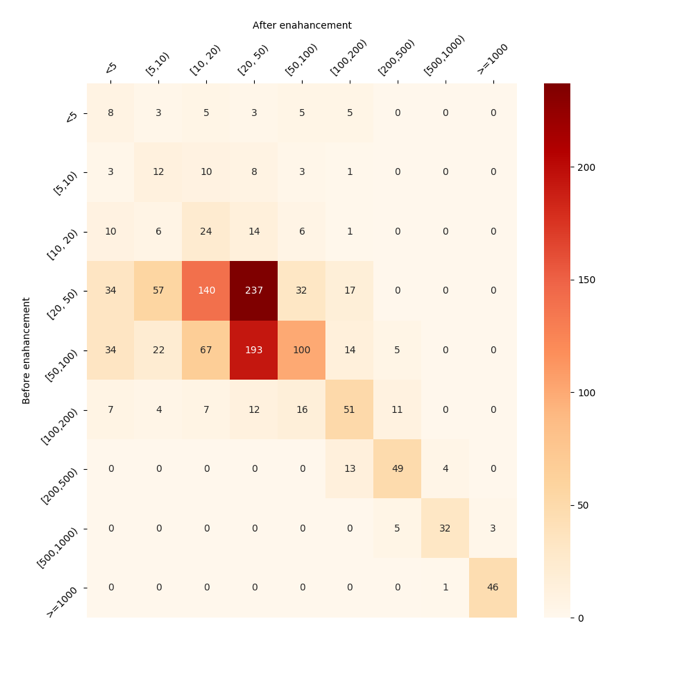


**HG002**

**(G) Enhancement using UNet on HG002 (H) Enhancement using CNN on HG002**


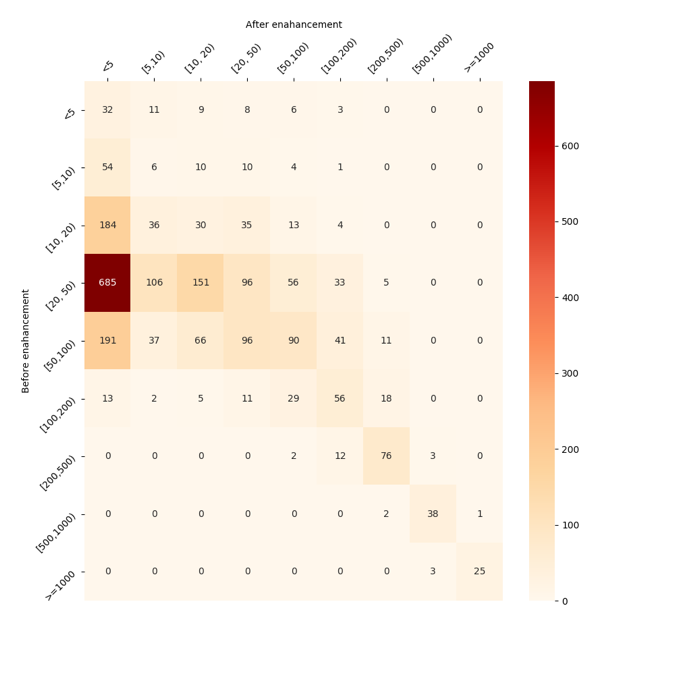

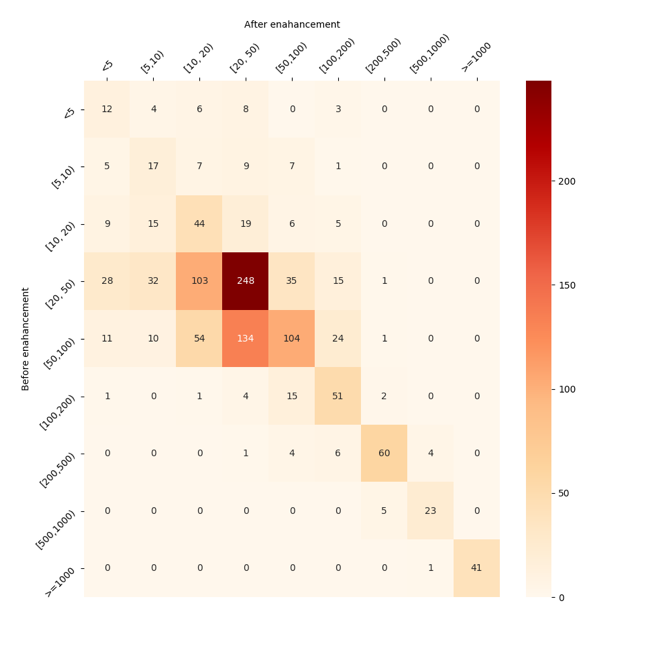

Supplement: S1 Fig — For NA12878 WGS data, (A), (C), (E), and (G) are the results of enhancement using UNet for NA12878, NA19238, NA19239, and HG002, respectively. (B), (D), (F), and (H) are the results of enhancement using CNN for NA12878, NA19238, NA19239, and HG002, respectively. (DOCX) [file pcbi.1009186.s006.docx]
