# Supplementary material for: Enhancing breakpoint resolution with deep segmentation model: A general refinement method for read-depth based structural variant callers
Source: PLoS Comput Biol. 2021 Oct 11;17(10):e1009186. doi: 10.1371/journal.pcbi.1009186 (PMC8504719; doi:10.1371/journal.pcbi.1009186)

**S2 Fig. Breakpoint change matrices of cross-sample enhancement on NA19238, NA19239 WGS data. For NA19238 WGS data, (A) and (B) are the results of enhancement using UNet and CNN, respectively. For NA19239 WGS data, (C) and (D) are the results of enhancement using UNet and CNN, respectively.**

**NA19238**

**(A) Enhancement using UNet on NA19238 (B) Enhancement using CNN on NA19238**


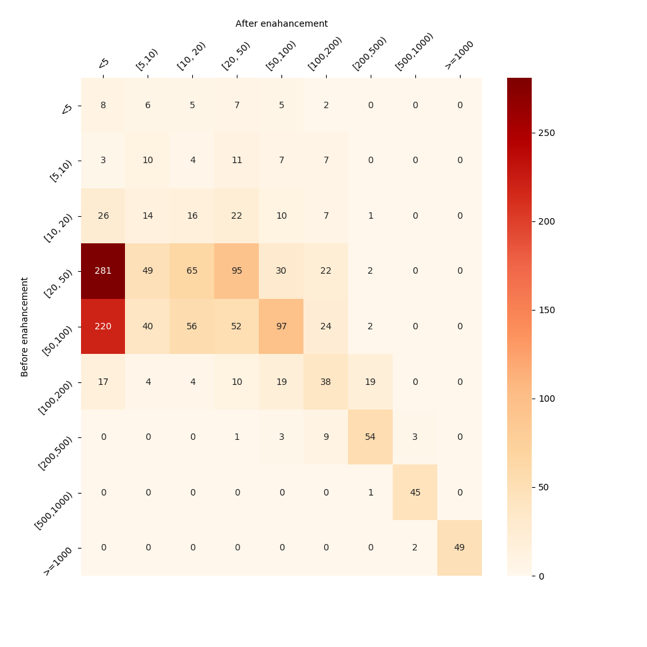

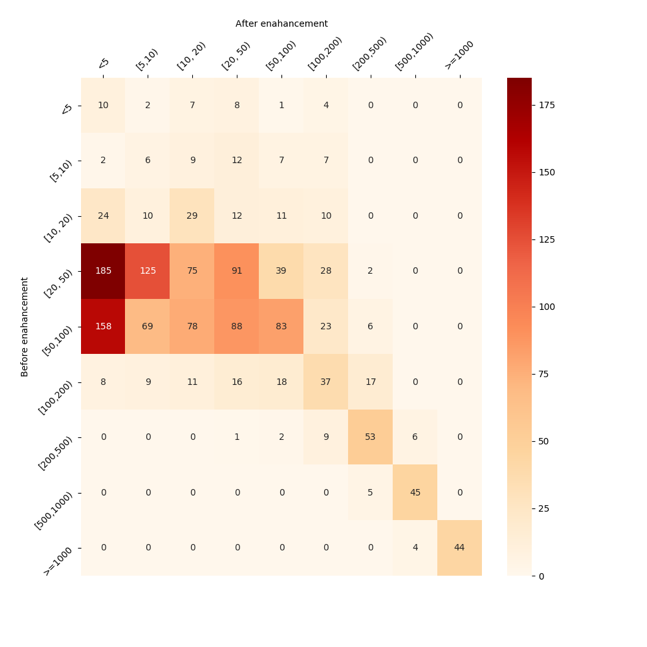


**NA19239**

**(C) Enhancement using UNet on NA19239 (D) Enhancement using CNN on NA19239**


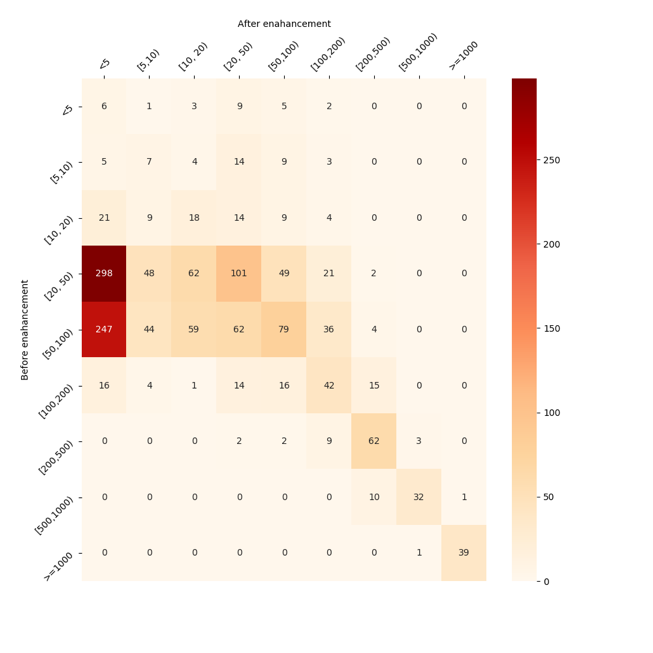

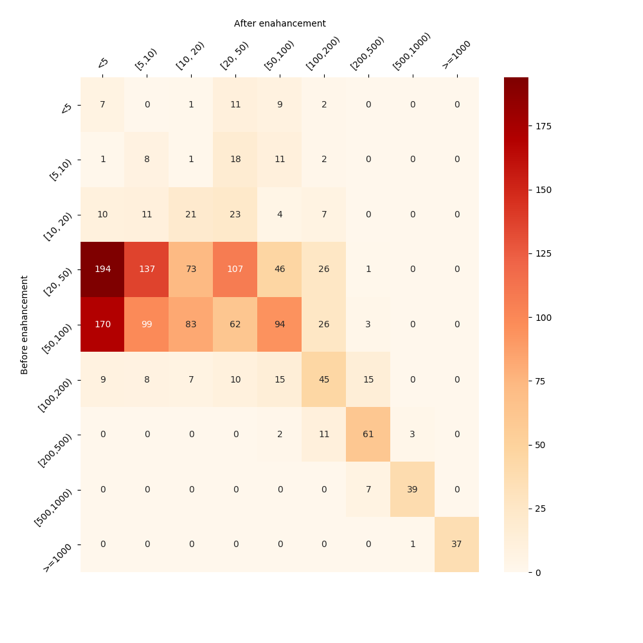

Supplement: S2 Fig — For NA19238 WGS data, (A) and (B) are the results of enhancement using UNet and CNN, respectively. For NA19239 WGS data, (C) and (D) are the results of enhancement using UNet and CNN, respectively. (DOCX) [file pcbi.1009186.s007.docx]
