# Supplementary figures and images for: Enhancing breakpoint resolution with deep segmentation model: A general refinement method for read-depth based structural variant callers
Source: PLoS Comput Biol. 2021 Oct 11;17(10):e1009186. doi: 10.1371/journal.pcbi.1009186 (PMC8504719; doi:10.1371/journal.pcbi.1009186)

**S3 Fig. Breakpoint change matrix of the UNet enhancement on COLO829 tumor WGS data.**


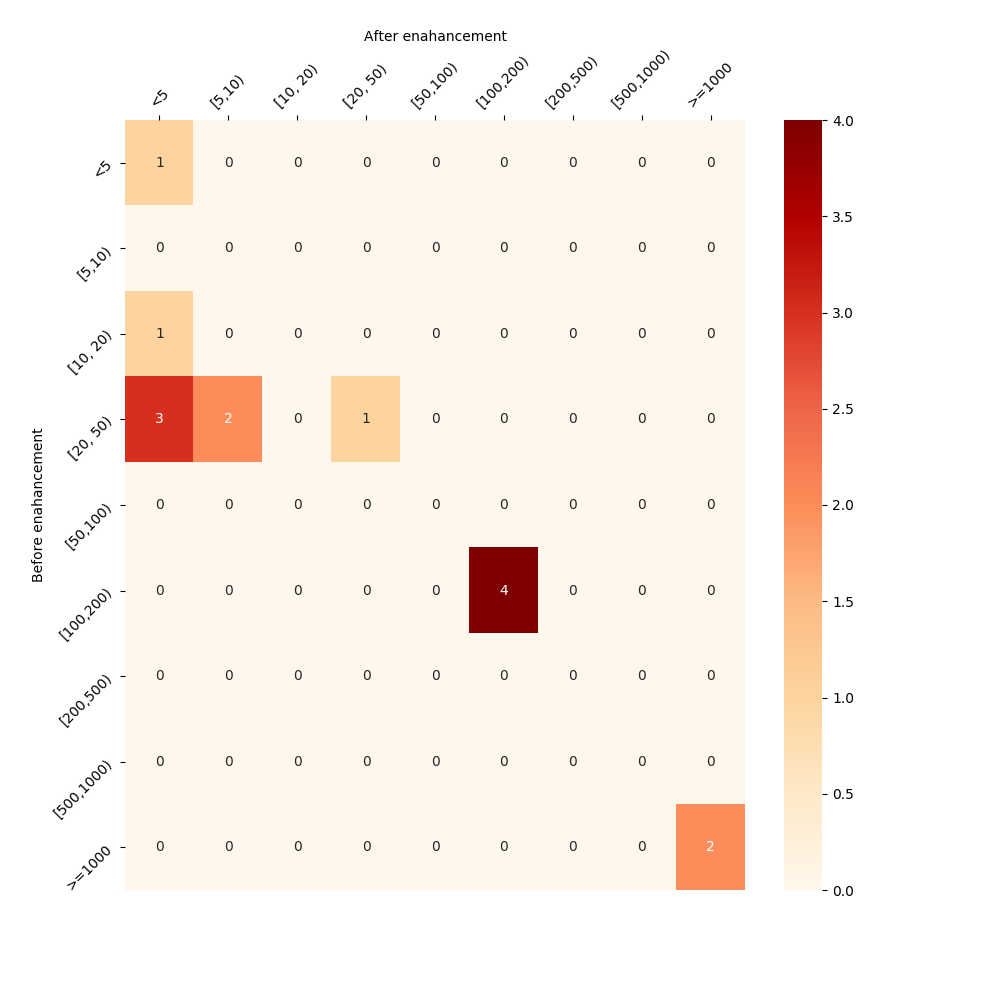

Supplement: S3 Fig — (DOCX) [file pcbi.1009186.s008.docx]
