# Supplementary material for: Enhancing breakpoint resolution with deep segmentation model: A general refinement method for read-depth based structural variant callers
Source: PLoS Comput Biol. 2021 Oct 11;17(10):e1009186. doi: 10.1371/journal.pcbi.1009186 (PMC8504719; doi:10.1371/journal.pcbi.1009186)

**S4 Fig. Segmentation results of specific cases on the simulated data. The dashed line indicates the position of the gold-standard breakpoint. Coordinates in red are the SV-overlapping coordinates.**

1. **Cases of positive enhancement by UNet.**

**obvious case less obvious case**

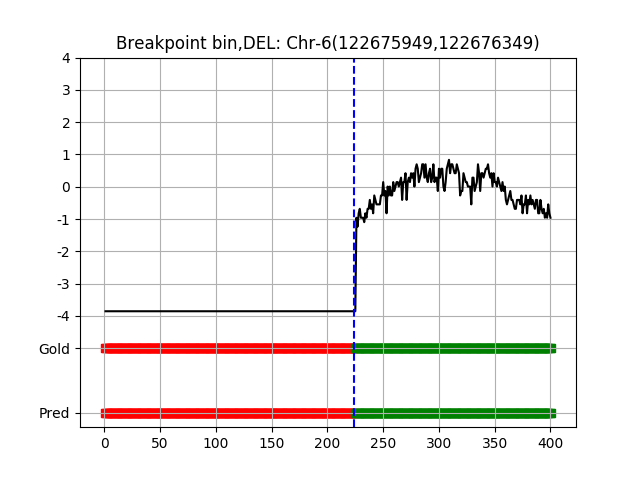

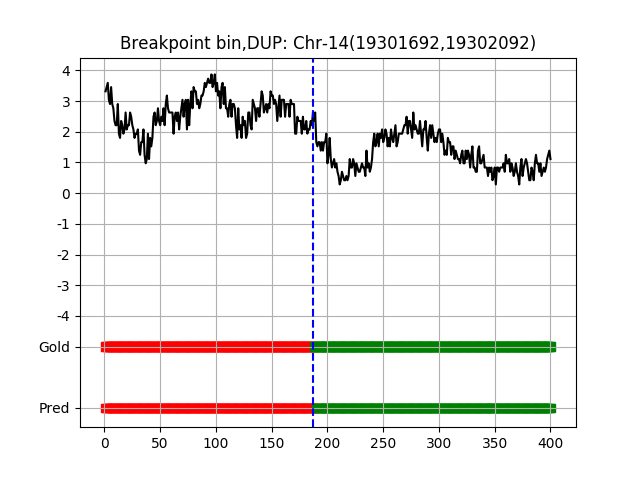


 **(B) Cases of small SVs entirely inside the screening window.**

**Unet segmentation**


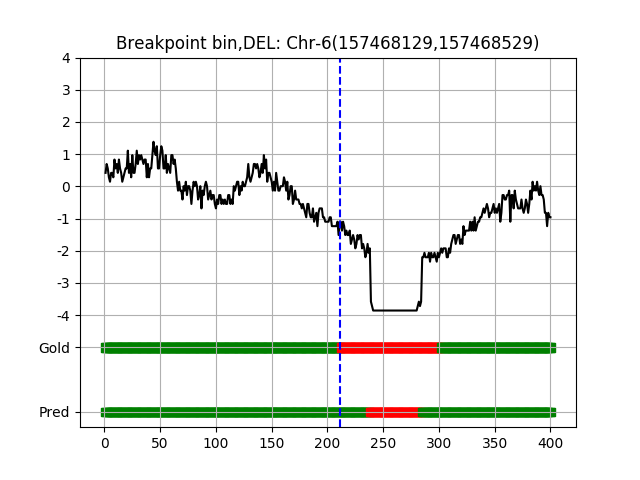

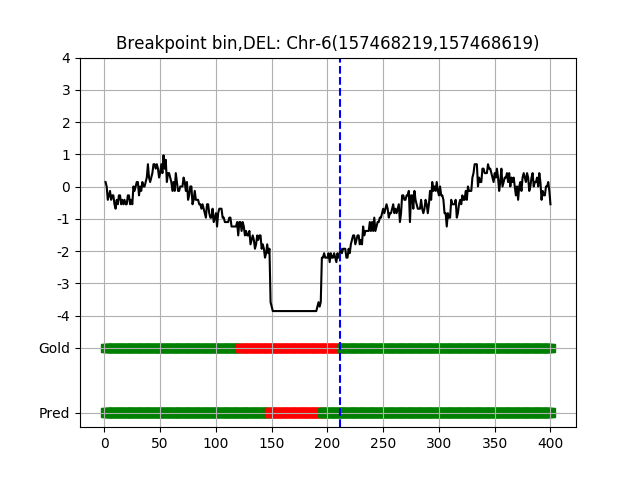


**Left Right**

**CNN segmentation**


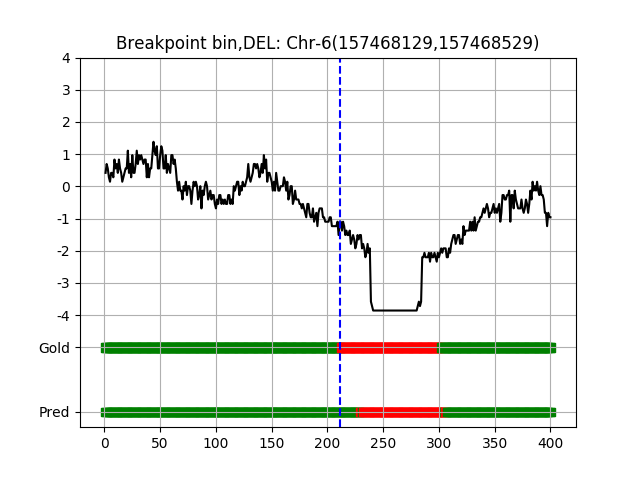

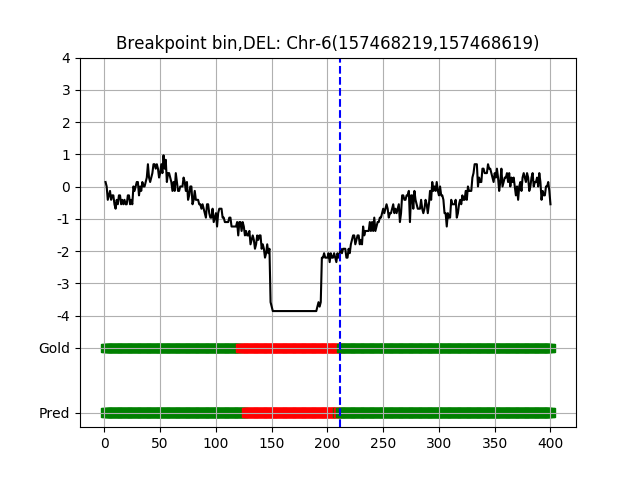


**Left Right**

**(c) Negative segmentation examples of UNet.**


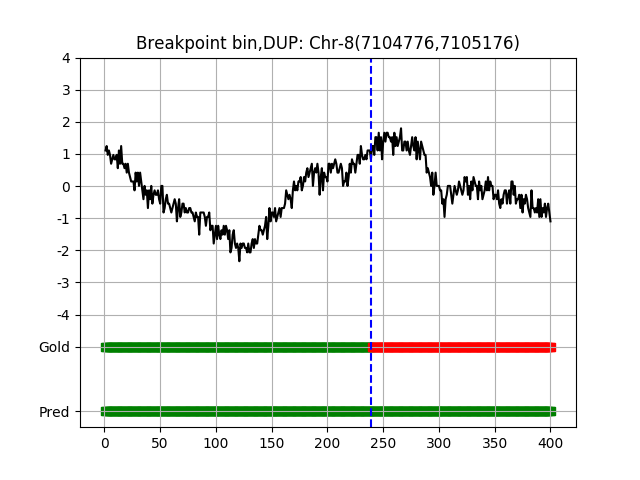

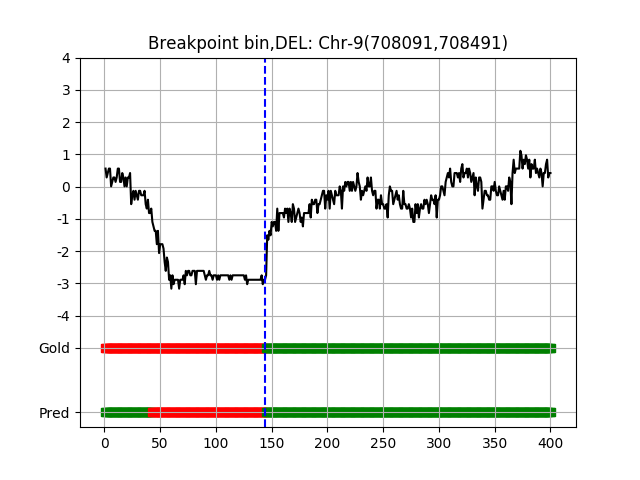

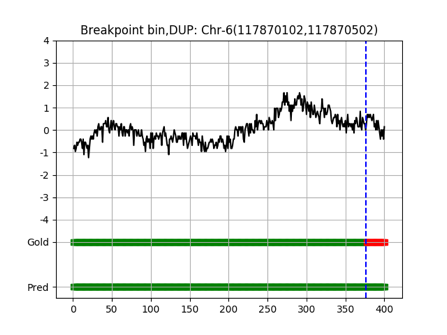

Supplement: S4 Fig — The dashed line indicates the position of the gold-standard breakpoint. Coordinates in red are the SV-overlapping coordinates. (A) Cases of positive enhancement by UNet. (B) Cases of small SVs entirely inside the screening window. (C) Negative segmentation examples of UNet. (DOCX) [file pcbi.1009186.s009.docx]
